# Fault Diagnosis of Rolling Element Bearings with a Two-Step Scheme Based on Permutation Entropy and Random Forests

**DOI:** 10.3390/e21010096

**Published:** 2019-01-21

**Authors:** Xiaoming Xue, Chaoshun Li, Suqun Cao, Jinchao Sun, Liyan Liu

**Affiliations:** 1 Jiangsu Key Laboratory of Advanced Manufacturing Technology, Huaiyin Institute of Technology, Huai’an 223003, China; 2 Faculty of Mechanical and Material Engineering, Huaiyin Institute of Technology, Huai’an 223003, China; 3 College of Hydropower and Information Engineering, Huazhong University of Science and Technology, Wuhan 430074, China

**Keywords:** fault diagnosis, rolling element bearing, permutation entropy, variational mode decomposition, statistical classification, random forests

## Abstract

This study presents a two-step fault diagnosis scheme combined with statistical classification and random forests-based classification for rolling element bearings. Considering the inequality of features sensitivity in different diagnosis steps, the proposed method utilizes permutation entropy and variational mode decomposition to depict vibration signals under single scale and multiscale. In the first step, the permutation entropy features on the single scale of original signals are extracted and the statistical classification model based on Chebyshev’s inequality is constructed to detect the faults with a preliminary acquaintance of the bearing condition. In the second step, vibration signals with fault conditions are firstly decomposed into a collection of intrinsic mode functions by using variational mode decomposition and then multiscale permutation entropy features derived from each mono-component are extracted to identify the specific fault types. In order to improve the classification ability of the characteristic data, the out-of-bag estimation of random forests is firstly employed to reelect and refine the original multiscale permutation entropy features. Then the refined features are considered as the input data to train the random forests-based classification model. Finally, the condition data of bearings with different fault conditions are employed to evaluate the performance of the proposed method. The results indicate that the proposed method can effectively identify the working conditions and fault types of rolling element bearings.

## 1. Introduction

The rolling element bearing is one of the most widely used components in rotating machines, and its running state has a direct relation to machinery reliability and safety. Common causes of bearing faults (race fault or ball fault) are overload, high temperature, assembly error and poor lubrication, and so on. Statistically, the bearing faults account for about 30% of all faults in rotating machines [1]. Therefore, the study of fault detection techniques and diagnosis methods of rolling bearings will be significant to promoting of the health condition of machines. In reviewing bearing fault diagnosis problems reported in literature, the collected vibration signals include plenty of state information related to the system’s dynamic characteristics. When a fault occurs, the impulse components can be effectively reflected by analyzing the corresponding vibration signal data. With the rapid development of the technology of transducers, communication and computer science, data-driven methods have become the mainstream for diagnosing the faults of rolling element bearings, since they do not require any prior knowledge about the parameters and mathematical model of diagnosis objects [2].

In general, the data-driven approach can be divided into three groups: the method of statistical analysis, the method of signal analysis, and artificial intelligence technology [3]. Some successful applications of statistical analysis methods in fault diagnosis have been reported in [4,5]. As an advanced signal processing technology, time-frequency analysis methods were widely used to extract important condition features from non-stationary signals for fault detection and diagnosis. Its developments and applications in fault diagnosis were reviewed in [6]. As for the first two methods, an expert’s rich knowledge will be required for their implementation and they also show some limitations in dealing with multi-fault diagnosis problems [5,6]. The machine learning method, as an important brand of artificial intelligence, has been widely applied in various fields, such as pattern recognition, fault diagnosis and data mining. Meanwhile, the related theories development provides great facility for the intelligent fault diagnosis technology [7]. It can be concluded that fault diagnosis based on artificial intelligence is a typical pattern recognition problem, where features extraction and classifier construction are the two crucial issues [8].

Due to the non-linear dynamic characteristics of bearings’ fault vibration signals, the traditional feature extraction methods based on Fourier transform and statistical analysis are no longer adequate for the effective and accurate diagnosis of bearing faults. Recently, various complexity measurement methods derived from information theory are proposed to characterize the vibration signals [9,10,11]. The widely used methods include energy entropy (EnE) [10,12], sample entropy (SE) [13,14], approximate entropy (ApEn) [15,16] and fuzzy entropy (FE) [17]. Although these methods have made certain achievements in the health monitoring and fault diagnosis of rotating machines, they still possess some deficiencies [8,11,18]. Permutation entropy (PE), as a non-linear dynamic parameter, was introduced by Bandit and Pompe to measure the randomness and dynamic mutation of time sequences [19]. Based on its advantages of intelligibility, low time consumption and strong robustness, PE has achieved many successful applications in the fault diagnosis of rotating machines [20,21,22,23].

In practice, however, vibration signals collected from complex systems usually possess non-linear and non-stationary properties and contain different signal components with multiscales. Most studies indicate that the PE feature with a single scale has certain limitations when describing the dynamic properties of vibration signals and some useful information riding in other scales may be ignored. To overcome this problem, multiscale permutation entropy (MPE) was proposed based on the coarse-grained processing of time series by Aziz and Arif [24]. However, the determination of the scale factor has a direct effect on the quality of MPE and it is often very difficult. More notably, the representation ability of MPE features could be weakened by the mixing components distributed in different frequency bands. To obtain an accurate description about signals, the entropy features extraction integrated with time-frequency analysis methods has become a hot spot in the fault diagnosis field. Some representative time-frequency analysis methods include wavelet transform (WT) [25], empirical mode decomposition (EMD) [26], local mean decomposition (LMD) [27] and variational mode decomposition (VMD) [28]. By using these methods, a complicated signal can be decomposed into a series of mono-components, and then the entropy features of mono-components can be extracted as features to reflect the local characteristics of the signals. For instance, wavelet packet decomposition (WPD)-based PE features were extracted to identify faults appearing in bearings [29]. Similarly, PE features combined with ensemble empirical mode decomposition were used to reveal the local characteristics of original signals under intrinsic time scales [30]. Unlike WT, the other three methods have removed the dependency to the base functions and indicate remarkable self-adaptability. Although EMD has been widely applied to signal processing and fault diagnosis [31], the sensitivity to noise and sampling frequency also limits its development. As a non-recursive signal decomposition method, VMD was proposed recently for adaptive signal decomposition [28]. In [32], the performance of VMD compared with LMD and EMD was discussed and the analysis results indicate that VMD has the advantages of high computation efficiency, strong robustness to background noise and accurate frequency band allocation. Hence, in this work, VMD-based PE is employed to extract fault features from vibration signals for working conditions identification.

After features extraction, the mode classifier should be established to accomplish the automatic fault diagnosis. Some machine learning-based diagnosis models, usually constructed by an artificial neural network (ANN), support vector machine (SVM), or extreme learning machine (ELM), have been reported in many works [33,34,35]. Compared with ANN and ELM, SVM is a kind of machine learning theory with excellent classification ability for small data sets developed on statistical learning theory. However, SVM also has certain limitations which have severely restricted its development in the fault diagnosis field. The parameters optimization by a grid search algorithm or other intelligent optimization algorithms is time intensive and needs large storage resources. In addition, the multiple binary classification models often should be exploited to solve the multi-class problem, resulting in high model complexity and low computational efficiency. In order to enhance the generalization performance and curb the overfitting problem of the single decision tree, the random forests (RF) classification method was put forward by Breiman [36], based on the concept of the bagging technique [37], CART algorithm [38] and features random selection [39]. Compared with the traditional decision tree algorithm, RF has manifested robust classification performance in solving high-dimensional and small-sample problems [40]. Furthermore, this method has also inherited the high interpretability capacity of the tree-based model. Currently, some successful applications can be found in text processing [41], speech recognition [42], face recognition [43], and pedestrian safety [44]. However, the applications of this method in fault diagnosis of rotary machine have been rarely reported [40,45]. Owing to its significant merits, the RF algorithm was employed to construct the automatic diagnosis model in this work.

When faced with complex and multi-class diagnostic problems, the diagnosis performance of models depends not only on the features and classification algorithms, but also on the diagnosis strategy. The traditional diagnosis model often is established based on the single-step diagnosis strategy, where this kind of models are trained by all training data once time with generating one classifier. Usually, the single step model is much more complicated to build and lacks consideration on the fault evolution law. In order to decrease the model complexity and enhance the flexibility of the diagnosis model, some diagnostic approaches with a better strategy have been reported recently. By extracting different fault features, a two-step approach was developed with integrating the statistical method and pattern recognition for plastic bearing fault diagnosis [5]. For rolling element bearings, some multistep diagnosis methods combined with permutation entropy and the time-frequency analysis method were proposed [30,46]. Compared with the method of literature [30], the diagnostic strategy of the literature [46] is more specialized and the threshold value of fault detection can be quantified by the statistical method. Although the two methods are able to perform well in diagnosis accuracy, the calculative efficiency of the SVM-based model still poses a significant impact on practical engineering applications.

The results of these studies indicate that stepwise diagnostic thought is more in accordance with the practical engineering problems and the human cognitive process. Hence, the stepwise diagnostic strategy was also employed in this work and the whole procedure was simplified into two steps: fault detection and fault identification. Considering features sensitivity in different stages, the single scale PE feature of the original vibration signals were employed for fault detection and the VMD-based PE feature were considered as input data to train the RF-based classification model. In the first step, a statistical classification model can be established on the basis of the PE distributions of the original signals, where the Chebyshev’s inequality was used to calculate the threshold value for the preliminary judgment of health condition. During the second stage, the classification ability of the VMD based PE features are easily weakened by the interference components or the components with less information. Benefiting from the random samples, the non-selected samples in the construction of each tree based model can be used to realize the bootstrap evaluation of the RF based model, where this procedure is called out-of-bag estimation [36]. This property can then be further used to measure the features importance. Here, the random forests method was employed twice in the second step. The first RF (1st-RF)-based model was established to calculate the importance degree of each scale PE. Next, the PE features in the scales with high importance were selected as the input data to train the second RF (2nd-RF)-based model for fault identification. Finally, the effectiveness and feasibility of the proposed diagnosis approach is verified by the bearing data set with different fault types and levels. The main contribution of this paper is the development of a two-step fault diagnosis method for rolling element bearings by using permutation entropy and random forests. Furthermore, with consideration of the own characteristics of feature data, a feature evaluation and selection method based on out-of-bag estimation is firstly proposed and discussed for the fault diagnosis of rolling element bearings.

The rest of the paper is organized as follows. Background knowledge about PE, VMD and RF is investigated in Section 2. In Section 3, the system framework of the proposed diagnosis approach is first introduced, and then the implementation procedures of features extraction, fault detection and fault identification are discussed. In Section 4, the proposed method is applied to the fault diagnosis of rolling element bearings and the analysis results are given. Conclusions are presented in Section 5.

## 2. Background Knowledge

### 2.1. Permutation Entropy (PE)

The principle of PE is based on the comparison of adjacent data without counting the specific data value of the time series. A brief description of PE is given as follows.

For a given time sequence of length *N*, {x(i),i=1,2,⋯,N}, its phase space can be reconstructed as

(1) $$\left\{ \begin{array}{c} X(1)=\{x(1),x(1+\lambda ),\cdots ,x(1+(m-1)\lambda )\}\\ \vdots \\ X(i)=\{x(i),x(i+\lambda ),\cdots ,x(i+(m-1)\lambda )\}\\ \vdots \\ X(N-(m-1)\lambda )=\{x(N-(m-1)\lambda ),x(N-(m-1)\lambda ),\\ ,\cdots ,x(N)\} \end{array}\right.$$

where *m* is the embedded dimension and λ is the time delay. As described in [19], the *m* data values in each *X*(*i*) can be arranged in ascending order as:

(2) $$X(i)=\{x(i+(j_{1}-1)\lambda )\leq x(i+(j_{2}-1)\lambda )\leq \cdots \leq x(i+(j_{m}-1)\lambda )\}$$

where $j_{d}$, d=1,2,⋯,m represents the column index of each element in the reconstructed component.

During the above sorting procedure, if any two or more element values are equal, their original orders are resorted by $j_{d}$’s value. For instance, if $x(i+(j_{p}-1)\lambda )=x(i+(j_{q}-1)\lambda )$ and $j_{p}<j_{q}$, the order of these two elements can be $x(i+(j_{p}-1)\lambda )\leq x(i+(j_{q}-1)\lambda )$. Hence, any reconstructed component *X*(*i*) can be mapped into a group of symbol sequences as:

(3) $$S(l)=\{j_{1},j_{2,}\cdots ,j_{m}\}$$

where l=1,2,⋯,k (k≤m!) and *m*! is the largest number of different symbol sequences. As described in Equation (3), *S*(*l*) is one of the *m*! symbol arrangement. Then the probability distribution of the each symbol sequence can be obtained and denoted as $P_{1},P_{2},\cdots ,P_{k}$, $\sum_{l=1}^{l=k}P_{l}=1$. By definition, the PE of {x(i),i=1,2,⋯,N} with *m* embedded dimension can be obtained as:

(4) $$H_{p}(m)=-\sum_{l=1}^{l=k}P_{l}\ln P_{l}$$

It can be noticed that *H_p_*(*m*) attains the maximum value ln(m!), when all the symbol sequences have the same probability distribution as $P_{l}=1/m!$. For convenience, the permutation entropy can be standardized as:

(5) $$H_{p}=H_{p}(m)/\ln(m!)$$

Obviously, the value of *H_P_* ranges from 0 to 1. A smaller *H_p_* value indicates that the time sequence is much more regular, and conversely, the larger the *H_p_* value, the more random the time series is.

### 2.2. Variational Mode Decomposition (VMD)

VMD is a newly developed variational methodology for adaptive decomposition of multi-component signals. Unlike EMD, it can non-recursively decompose a real valued input signal *f* into a collection of quasi-orthogonal intrinsic mode functions $\left\{u_{k}\}:=\{u_{1},u_{2},\cdots ,u_{K}\right\}$ with specific sparsity properties [28]. Each principal mode is compact around a center pulsation $\omega_{k}$ that is adaptively determined along with the decomposition and its bandwidth is estimated through the squared *L*^2^-norm of the gradient, so these composition modes are called band-limited intrinsic mode functions (BLIMFs). The solution of VMD that is constructed as a constrained variational problem can be expressed as follows:

(6) $$\underset{\left\{u_{k}\},\{\omega_{k}\right\}}{\min}\left\{\sum_{k}^{K}{\left\|\partial_{t}\left[\left(\delta (t)+\frac{j}{\pi t}\right)*u_{k}(t)\right]e^{-j\omega_{k}t}\right\|}_{2}^{2}\right\}\;s.t.\;\sum_{k}^{K}u_{k}=f$$

The constrained problem of Equation (6) can be addressed by introducing a quadratic penalty term α and Lagrangian multipliers λ. The augmented Lagrangian thus can be expressed as follows:

(7) $$\begin{array}{cc} L\left(\{u_{k}\},\{\omega_{k}\},\lambda \right) & =\alpha {\sum_{k}\left\|\partial_{t}\left[(\delta (t)-\frac{j}{\pi t})*u_{k}\right]e^{-j\omega_{k}t}\right\|}_{2}^{2}\\ & +{\left\|f(t)-\sum_{k}u_{k}(t)\right\|}_{2}^{2}+\langle \lambda (t),f(t)-\sum_{k}u_{k}(t)\rangle . \end{array}$$

The saddle point of Equation (7), which is the optimal solution of the constrained problem in Equation (6), can be obtained by using the alternate direction method of multipliers (ADMM). The detailed realization of VMD is summarized with pseudo-code form as follows (Algorithm 1):

**Algorithm 1** VMD Realization  Initialize $\{u_{k}^{1}\},\{\omega_{k}^{1}\},\lambda^{1},n=0$, boolean = ture  while *Boolean*  *n* = *n*+1  for *k* = 1:*K*(8)$$\;u_{k}^{n+1}=\underset{u_{k}}{\arg\min}L\left(\{u_{i<k}^{n+1}\},\{u_{i\geq k}^{n}\},\{\omega_{i}^{n}\},\lambda^{n}\right)$$  end  for *k* = 1:*K*(9)$$\;\omega_{k}^{n+1}=\underset{\omega_{k}}{\arg\min}L\left(\{u_{i}^{n+1}\},\{\omega_{i<k}^{n+1}\},\{\omega_{i\geq k}^{n}\},\lambda^{n}\right)$$  End(10)$$\;\lambda^{n+1}=\lambda^{n}+\tau (f-\sum_{k}^{K}u_{k}^{n+1})$$  if $\sum_{k}^{K}({\left\|u_{k}^{n+1}-u_{k}^{n}\right\|}_{2}^{2}/{\left\|u_{k}^{n}\right\|}_{2}^{2})<\varepsilon$   *boolean* = false  end

During the decomposition process, the update of $u_{k}$, $\omega_{k}$ and λ can be solved in spectral domain. The quadratic problem in Equation (8) solved as:

(11) $${\hat{u}}_{k}^{n+1}(\omega )=\frac{\hat{f}(\omega )-\sum_{i\neq k}{\hat{u}}_{i}(\omega )+\frac{\hat{\lambda }(\omega )}{2}}{1+2\alpha {\left(\omega -\omega_{k}\right)}^{2}}$$

The minimization problem in Equation (9) can be easily solved as:

(12) $$\omega_{k}^{n+1}=\frac{\int_{0}^{\infty }\omega \left|{\hat{u}}_{k}{\left(\omega \right)}^{2}\right|d\omega }{\int_{0}^{\infty }\left|{\hat{u}}_{k}{\left(\omega \right)}^{2}\right|d\omega }$$

In the spectral domain, the update of λ can be obtained as:

(13) $${\hat{\lambda }}^{n+1}(\omega )\leftarrow {\hat{\lambda }}^{n}(\omega )+\tau \left(\hat{f}(\omega )-\sum_{k}{\hat{u}}_{k}^{n+1}(\omega )\right)$$

### 2.3. Random Forests (RF)

#### 2.3.1. Fundamental Principle

**Figure 1:**
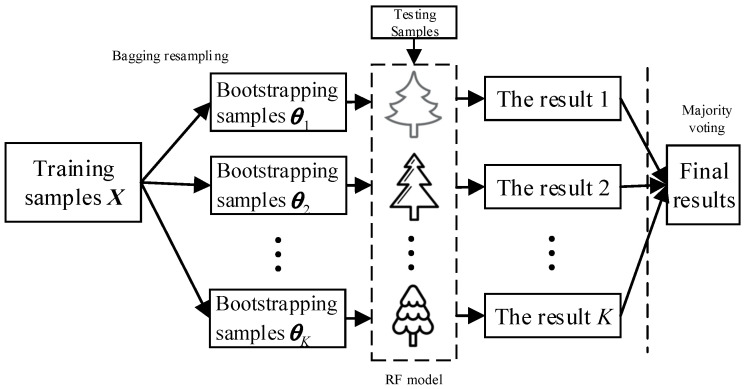
The typical classification model of random forests.

Random forests (RF) composed of multiple decision trees $\left\{h(a,\theta_{k}),k=1,2,\ldots ,T\right\}$ is one kind of ensemble classifier, where *T* denotes the total number of decision trees, $\left\{\theta_{k}\right\}$ are the random vector set with independent and identical distribution and a is the random inputted feature vector. The final classification result of RF is obtained based on the majority voting method or the average output of all decision trees. Its classification model is shown in Figure 1. Compared with the decision tree analysis, random forests possesses better generalization performance and inhibition capacity of overfitting by aggregated multiple CART trees [36]. The algorithm flow is given as follows.

(1) Get Bootstrapping samples $\theta_{k}$ by Bagging method from the training set *X*. Specifically, the $\theta_{k}$ is obtained by random selection with replacement, where the sample sizes of $\theta_{k}$ and *X* are identical.

(2) For each $\theta_{k}$, based on the CART algorithm, establish the binary tree according to the following steps.

① Suppose $\theta_{k}$ has *M* characteristics, and randomly select *m* alternative features from the whole characteristics at the branch growth of each node. Throughout the forests construction, the value of κ is identical.

② According to the Gini impurity minimization, obtain the best split attribute from the *m* characteristic to enable the binary tree develop completely, where pruning is not performed on trees.

(3) Repeat steps (1) and (2) until *T* decision trees were constructed, and the classification model of random forests was achieved.

(4) For unknown samples, the final classified results can be calculated by employing the majority voting method, as shown in the following formula,

(14) $$c=\arg\max_{c}(\frac{1}{T}\sum_{k=1}^{T}I(h(a,\theta_{k})=c))$$

where I(·) is indicator function and *c* represents the sample type with the most votes.

For all testing samples, the voting results can be represented by the chaotic matric *CM*, where the element *CM*(*i*, *j*) denotes the voting numbers with *j*-th type of the *i*-th type sample for all decision trees and *I* = *j* represents the correct classified result. The final classification accuracy *CA* is:

(15) $$CA=\sum_{i=1}^{M}CM(i,i)/\sum_{i,j=1}^{M}CM(i,j)$$

It can be concluded that the numbers of random attributes and decision trees have a great importance to the model accuracy and calculation cost. Generally, κ equals to $\sqrt{M}$ and the value of *T* ranges from 500 to 1000. The complete algorithm of RF in detail can be found in [36].

#### 2.3.2. Importance Evaluation of Characteristics Based on Out-of-Bag (OOB) Estimation

Through analysis, it can be found that the probability of remaining selection of each sample is ${\left(1-1/n\right)}^{n}$ during the process of $\theta_{k}$ construction. By calculation, if the sample size is big enough, ${\left(1-1/n\right)}^{n}$ is roughly equal to 0.368. The samples with remaining selection in the construction of each tree are called out-of-bag (OOB) data, and the model evaluation by OOB data is defined as OOB estimation [47].

For the established forests, one set of out-of-bag data OOB*_k_* obtained from the *k*-th decision tree were employed to evaluate the model performance and then the corresponding classification error rate can be obtained. Complying with the same pattern, the classification error rates of all decision trees can be obtained and the average value of all classification error rates is considered as the generalization error of random forests, as well as the classification performance estimation of the model.

Some research indicates that the OOB estimation is unbiased and this method can not only enhance the evaluation efficiency of the classification model, but also the result is consistent with that of cross validation [36]. Based on the randomness of features selection, the OOB estimation can be employed to measure the importance of each feature for fault representation. The specific steps are given as follows:

(1) For a given random forests classification model, perform evaluation tests with all OOB data and calculate the average of correct classification rates, denoted as $\bar{CA}$.

(2) Randomly change the values of the *i*-th characteristic in all OOB data, and recalculate the average value of the correct classification rates of the new OOB data, which is denoted as $\bar{CA_{i}}$. The difference value of $\bar{CA}$ and $\bar{CA_{i}}$ can be expressed as $dif_{i}=\bar{CA}-\bar{CA_{i}}$.

(3) Repeat step (2) as many times as the difference values in terms of all attributes are obtained, and they are denoted as $DIF=\{dif_{1},dif_{2},\cdots ,dif_{M}\}$.

Obviously, the value of $dif_{i}$ can reflect the effects of the corresponding feature on classification accuracy, where the feature with larger $dif_{i}$ means it has a better differentiation capacity on fault types than the one with smaller $dif_{i}$. Hence, the $dif_{i}$ index can be employed to evaluate the importance of one feature on fault representation. In the next sections, this peculiarity was utilized to refine the original feature sets.

## 3. The Proposed Fault Diagnosis Model

**Figure 2:**
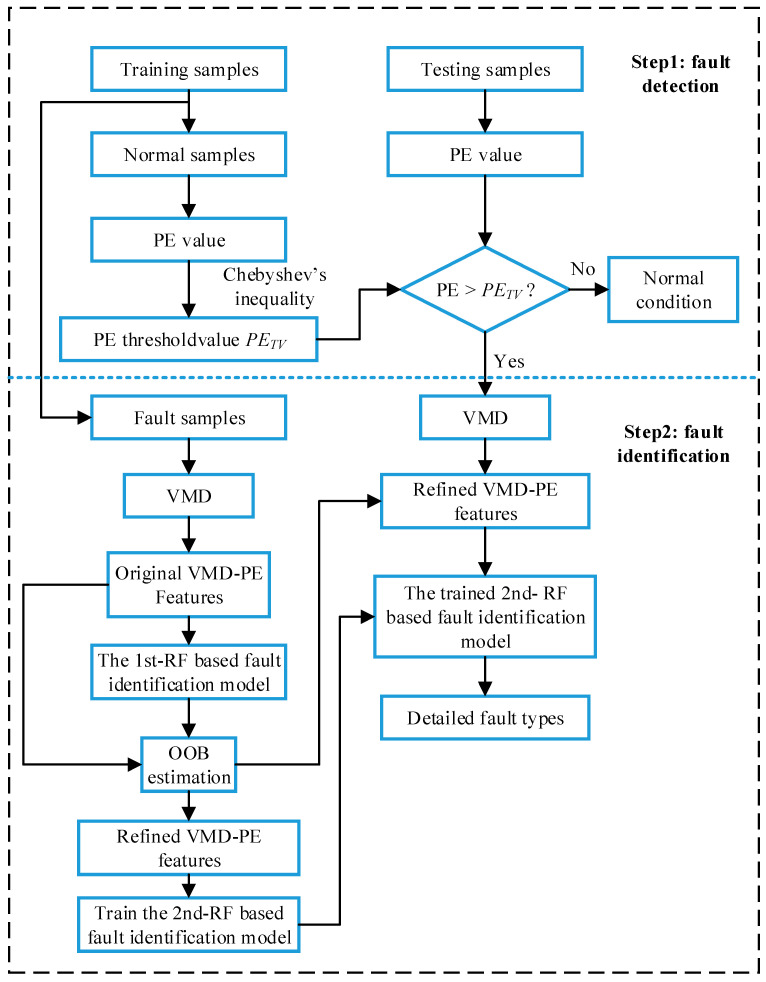
System framework of the proposed model.

According to the fault evaluation law of rolling element bearing and the human intelligence way, a two-step fault diagnosis scheme that included fault detection and fault identification was proposed in this work. As shown in Figure 2, a statistical classification model and one fault classifier based on random forests were constructed respectively in different steps. In the first step, the statistical distributions of the permutation entropy of vibration signals can be obtained based on historical status data analysis, and then it was used to realize preliminary judgment of the equipment health condition. In the next step, the specific fault types were identified by employing the random forests-based diagnostic model where the VMD-PE features were considered as inputted data. Furthermore, the importance of each scale PE feature was evaluated by OOB estimation and the original VMD-PE features were refined. The detailed implementation of each stage will be explained next.

### 3.1. Features Extraction Based on PE and VMD

As shown in Figure 2, two types of bearing condition features, single scale PE features and VMD-based PE features are extracted from original signals and BLIMFs respectively to identify bearing working conditions in different steps.

In the first stage, how to succeed in correctly and timely detection of fault occurrence is the prior task for engineers and technical staff. When faults occur, the spatial characteristics of vibration signals will be transformed compared with the normal condition. According to the above analysis, the permutation entropy can be utilized to magnify the weak variation of time sequences and it is an effective method to detect the mutation of signals. Hence, the permutation entropy feature *PE_i_* of one given signal *x_i_* can be extracted to realize the health judgment.

Generally, the characteristic of fault signals is much more complicated than that of the normal signals because of friction, vibration, load, fatigue damage and other factors. As a result, it can lead to a poor description ability of the single scale PE feature on fault identification. In this work, the VMD-based PE features are employed to measure the randomicity and dynamic mutation of fault signals and the features are considered as characteristic parameters for the second step. The calculation of VMD-PE is provided as follows.

(1) For a given signal *x_i_*, obtain a collection of BLIMF components $\left\{u_{k}\}=\{u_{1},\cdots ,u_{K}\right\}$ by VMD. During signal decomposition, the quadratic penalty α and the bandwidth τ are respectively set to the default values of 2000 and 0.01.

(2) Calculate the permutation entropy value for each BLIMF component and obtain the multi-scale permutation entropy feature of the original signal *x_i_*, denoted as $MPE_{i}=\{MPE_{i1},\cdots ,MPE_{iK}\}$, where *MPE_ij_* is the permutation entropy value of the *j*-th BLIMF.

### 3.2. Fault Detection Based on the Statistical Classification Model

In reality, fault early warming is very important for the prevention of fault expansion. When bearing faults occur, the high-frequency impulse parts motivated by faults make the vibration signals more complex. According to related theory, the PE values of fault signals are larger than that of normal signals, and this property can be employed to detect early faults [30]. Consequently, when the statistical classification method is used to judge the bearing health condition, how to determine the threshold value in terms of *PE* distributions becomes an important issue.

Generally, the vibration data of bearings with a normal condition are much easier to obtain than that data with fault conditions. Meanwhile its distributions of *PE* value are more regular and can be discussed more easily. Hence, the inspecting data with normal condition is employed to determine the threshold value of PE. In reality, the PE distribution of a normal condition does not always strictly obey normal distribution. Therefore, the statistical knowledge of the normal distribution cannot be utilized to calculate the threshold value. According to the probabilistic statistics, Chebyshev’s inequality can be employed to analyze any unknown distribution. The probability distribution regularities of PE values of normal bearings can be obtained by Chebyshev’s inequality as

(16) $$P(\left|PE-\mu \right|\geq \varepsilon \sigma )\leq \frac{1}{\varepsilon^{2}}$$

where μ and $\sigma^{2}$ are the mean value and variance of PE values of normal bearings respectively, and the probability upper bond $1/\varepsilon^{2}$ is actually the false alarm rate. It means that the probability of the PE values of normal condition with greater than μ+εσ should be no more than $1/\varepsilon^{2}$. Here, the upper bond μ+εσ can be set as the threshold value. Let *PE*(*i*) denotes the *i*-th data point of PE. Then, for *N* samples, its health judgment threshold value can be obtained as:

(17) $$PE_{TV}=\frac{1}{N}\sum_{i=1}^{N}PE(i)+\varepsilon \sqrt{\frac{1}{N-1}{\left[\sum_{i=1}^{N}\left(PE(i)-\frac{1}{N}\sum_{i=1}^{N}PE(i)\right)\right]}^{2}}$$

In general, the false alarm rate $1/\varepsilon^{2}$ is set to 0.05 or 0.01. Practically, the value of $1/\varepsilon^{2}$ is empirically determined with the specific objects. If the alarm rate is relative large, resulting in a relative small value of $PE_{TV}$, the misdiagnosis probability of normal samples can be heightened. Conversely, the probability may be reduced. In this paper it is set to 0.05, and then the $PE_{TV}$ can be computed as:

(18) $$PE_{TV}=\frac{1}{N}\sum_{i=1}^{N}PE(i)+4.4721\sqrt{\frac{1}{N-1}{\left[\sum_{i=1}^{N}\left(PE(i)-\frac{1}{N}\sum_{i=1}^{N}PE(i)\right)\right]}^{2}}$$

### 3.3. Random Forests-Based Fault Identification

In the fault detection stage, if the PE value of one signal is large than *PE_TV_*, it may be assumed that there are faults in bearings. Considering the type diversity and characteristic complexity of bearing faults, this work proposed an intelligence diagnostic model based on VMD-PE and RF. To further enhance the diagnostic performance of the model, the original VMD-PE features were reelected by using OOB estimation, and then the refined VMD-PE features are considered as the inputted data for training model. The implementation of the second step is described as follows.

(1) After the first step, decompose the signal with fault conditions into a series of BLIMF components and obtain the VMD-PE features.

(2) Input the original VMD-PE feature set and train the first RF (1st-RF) model, where the 1st-RF model is constructed with the original VMD-PE features and can mainly be utilized for the refined selection of VMD-PE features.

(3) Based on the 1st-RF model, reelect the original VMD-PE features by OOB estimation. Then, choose the first several scale PE features with larger importance as the optimized feature set.

(4) Construct the second RF (2nd-RF) model based on the refined VMD-PE features and obtain the final diagnostic model.

(5) Input the testing samples and identify the corresponding fault types.

As illustrated in Figure 2, the diagnostic procedure of the two steps is not independent, where the diagnosis results of the last step has direct effects on the subsequent step. The diagnosis results of the second step are calculated based on the assumption that the diagnostic accuracy of the precious step is 100%. In order to explain and analyze the performance of the proposed model, the diagnosis accuracy of the testing samples is calculated by the following method: obtaining the sample numbers of wrong results in two steps respectively; and calculating the total error rate of the whole diagnosis procedure. Let *E*_1_ denotes the diagnosis error rate of the first step, and *E*_2_ be the error rate of the fault identification step. Hence, the final diagnosis accuracy η of the proposed model can be calculated as:

(19) $$\eta =(1-\frac{E_{1}*H+E_{2}*h}{H})\times 100\%$$

where *H* is the total number of the testing samples and *h* is the total number of testing samples with fault conditions.

## 4. Experimental Results and Analysis

**Figure 3:**
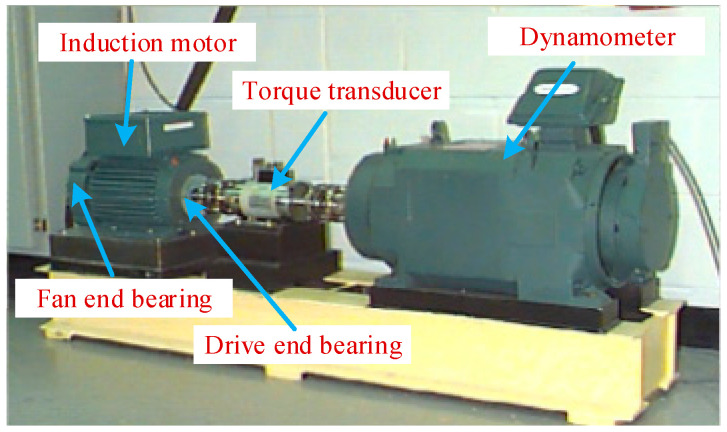
Fault test platform of rolling element bearings.

In this section, the experimental data freely provided by Bearing Data Center of Case Western Reserve University is employed to validate the proposed diagnosis method. As shown in Figure 3, the experimental setup consists of a 2 hp Reliance Electric motor (left), a torque transducer (middle) and a dynamometer (right) [47]. The testing bearings installed on both ends of the motor housing support the motor shaft, where the type of the testing bearings is the 6205-2RS deep groove ball bearing (SKF, Gothenburg, Sweden). Different faults (inner race fault, outer race fault or ball fault) were separately seeded on the normal bearings by an electric discharge machine and then faulted bearings were reinstalled into the test motor. Four defect sizes (0.0178 cm, 0.0356 cm, 0.0533 cm and 0.0711 cm) were processed on the inner race fault and ball fault bearings, and three defect sizes (0.0178 cm, 0.0356 cm, 0.0533 cm) were processed on the outer race fault bearings. Here, vibration data under different fault conditions were collected from the acceleration sensor installed on the drive end of the motor housing with different loads of 0 to 3 hp (motor speeds of 1797 to 1720 rpm). The signal sampling frequency was 12 kHz, and the data length of each working condition was 120,000 points.

Here, vibration data of the drive end only under loads of 0hp (1797 rpm) and 2hp (1750 rpm) were considered, and each load condition contains 12 working conditions, as illustrated in Table 1. To perform fault diagnosis, the measured data under each working condition was divided averagely into 40 sub-signals. In the first stage, the 50% of the samples with normal condition were randomly selected to determine the threshold value *PE_TV_*. Then, in the second step, the MPE feature sets with fault condition were split into two groups: 25% and 75%. 25% was employed as training data to train the RF-based fault identification model and the remaining 75% was used to test model.

**Figure 4:**
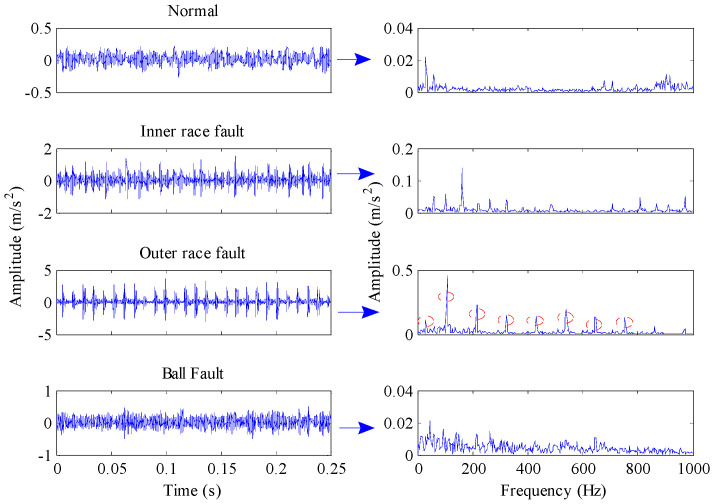
Time domain waveforms and the corresponding envelope spectrums of the vibration signals under different working conditions.

**Figure 5:**
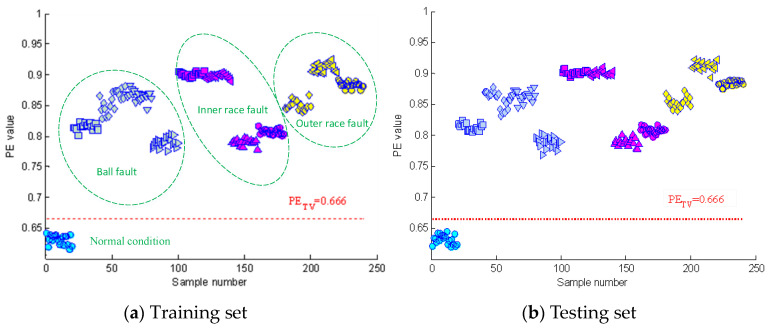
The PE distribution of the vibration signals of Case 1.

**Figure 6:**
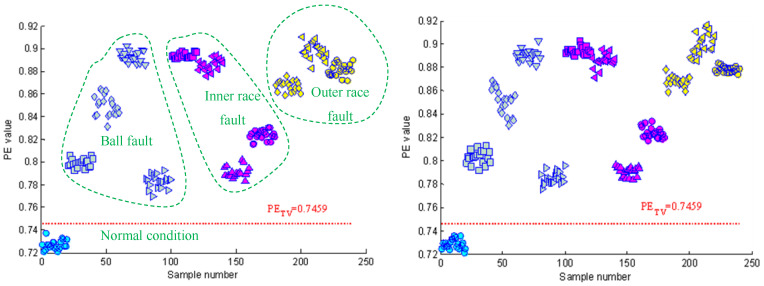
The permutation entropy (PE) distribution of the vibration signals of Case 2.

Figure 4 shows the time domain waveforms of the vibration signals with different fault conditions and the corresponding frequency spectrograms. As can be seen from the figure, the spectrograms of fault signals have significant impact frequencies and their spectral characteristics are more complex than that of normal signals. Section 2.1 describes how permutation entropy possesses effective measurement capacity of signal complexity. Thus, this point can be tentatively utilized to detect incipient faults of rolling element bearings. The permutation entropy distribution of training samples of Case 1 is displayed in Figure 5a. It can be seen from this figure that the permutation entropy values of fault signals clearly outweigh the values of normal signals. So, how to calculate the threshold value to detect incipient faults is an important issue. According to Equation (18), the threshold value of Case 1 becomes 0.666. The red dotted lines in Figure 5 represent the threshold value calculated by the PE values of normal condition signals. As shown in Figure 5, the fault conditions can be separated from normal condition by the statistical classification method with 100% accuracy. Also, the health judgment accuracy of Case 2 is also 100%, as shown in Figure 6. 

**Figure 7:**
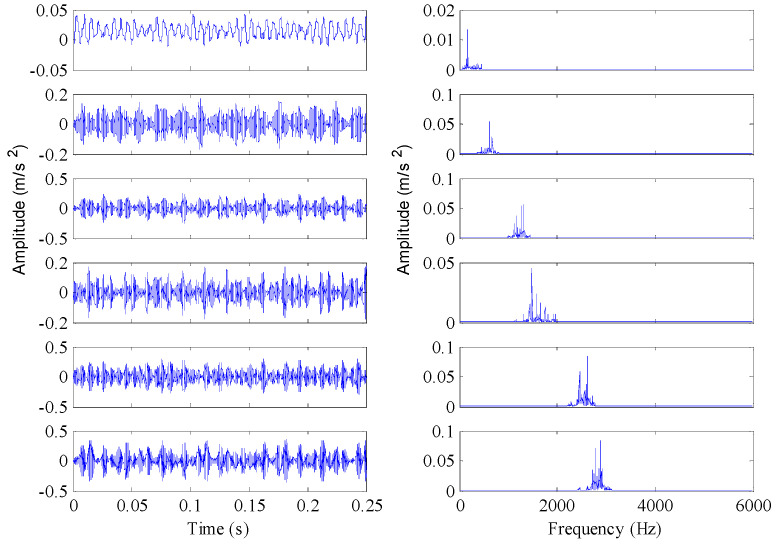
Decomposed results obtained by variational mode decomposition (VMD) and envelope spectrums of the corresponding band- limited intrinsic mode function (BLIMF) components.

**Figure 8:**
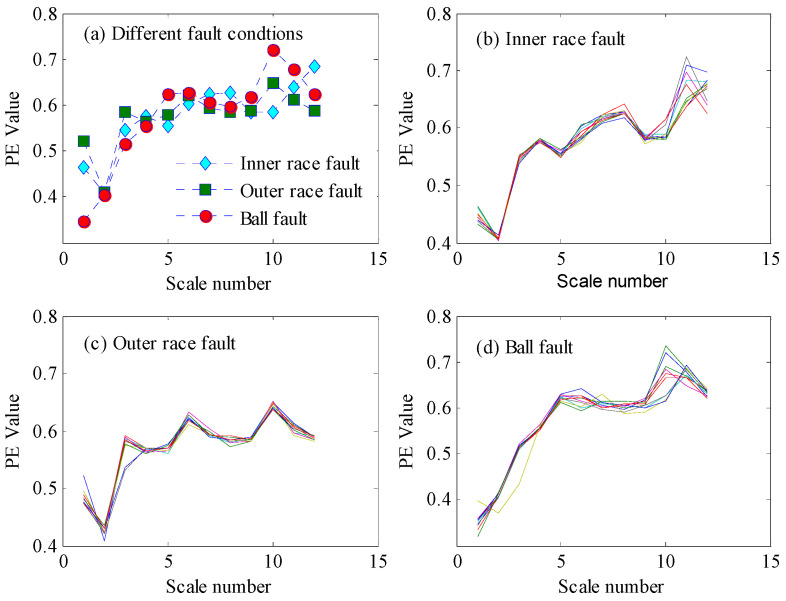
Dissimilarity and aggregation of the VMD-PE distributions under different fault conditions.

Although the PE features perform well in detecting faults, the detailed fault types cannot be classified only based on the single-scale PE features. As shown in Figure 5 and Figure 6, there are serious aliasing regions of the PE values with different fault conditions in the vertical direction. In the fault identification stage, the PE features of each BLIMF component were extracted to construct the fault classifier and to attain the final diagnosis results. Next, the vibration signals were decomposed by VMD with *K* = 12, where *K* can be determined based on the observation of the center frequency of each BLIMF [48]. Figure 7 gives the decomposed results of one vibration signal with inner race fault and the frequency spectrogram of each BLIMF. For convenience sake, only the first six BLIMF components were displayed in Figure 7. Clearly, the original complicated signal can be decomposed into a series of mono components with relatively single frequency information. So the frequency characteristics contained in the original fault signals can be easily extracted from each BLIMF component. As a result, multiscale PE features extracted from each BLIMF component can provide a more abundant description of the dynamic characteristics of the vibration signals compared with the single scale PE features. The distribution dissimilarity of the VMD-PE features with different fault types and the aggregation of the VMD-PE with the same fault type are displayed in Figure 8. As shown in Figure 8a, the VMD-PE performs good differentiability for different fault conditions. Meanwhile, the VMD-PE distributions with same fault type performs good clustering, as illustrated in Figure 8b–d, where the lines with different colors in each figure represent the variation trend of VMD-PE values of different signal samples with the same fault type. The results demonstrate that VMD-PE is one kind of feasible fault features for bearings fault identification.

After VMD-PE features extraction, the total VMD-PE features set were randomly divided into two parts: 25% and 75%. For comparison, the training samples were used to train the RF-based, SVM-based and ELM-based fault classifier respectively, where the other 75% samples were employed as testing data to evaluate the performance of each diagnosis model. To improve the reliability of the experiment results, each diagnosis experiment with different classification algorithms was performed repeatedly 20 times based on the same computing platform. Table 2 gives the diagnosis results of testing samples for different diagnostic models. The mean diagnosis accuracy (%) and the standard deviations (%) are given in the second and fourth columns of Table 2, while the mean cost time (s) is reported in the third and fifth columns. 

As shown in Table 2, although the ELM-based model requires a minimum of computation time, its diagnosis accuracy is the lowest compared with other two methods. Meanwhile, the diagnosis accuracy of SVM is relative high but it is time consuming. It is clearly that these two methods cannot do well in both of the aspects of efficiency and precision. The diagnosis results of the RF-based model were also listed in Table 2, where the average precisions of two cases are 98.44% and 99.09%, respectively and the computation time is much less than that of the SVM-based model. Diagnosis results indicate that the RF-based model performs well in both efficiency and precision, which means it is a powerful classification algorithm for fault diagnosis of rolling element bearings.

**Figure 9:**
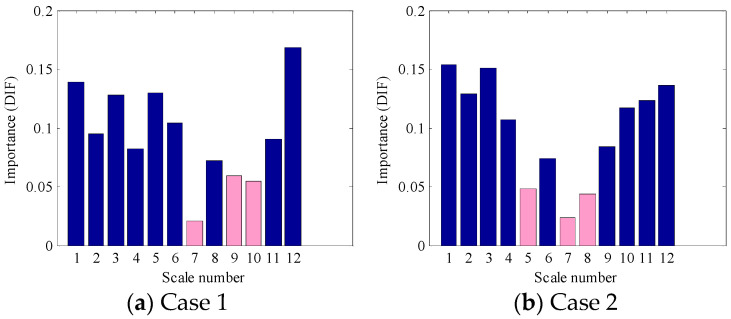
Importance evaluation of multiscale permutation entropy (MPE) features based on out-of-bag (OOB) estimation.

As displayed in Figure 7, each BLIMF contains certain frequency information, but it does not mean that each component could play a positive role in fault identification. Actually, the interference components or the components with less condition information will seriously block the proper identification of faults and are hard to distinguish and eliminate. In this work, the OOB estimation was used to evaluate the PE features importance of each BLIMF component to further refine the original VMD-PE set. The importance value of each scale PE feature is displayed in Figure 9, where the PEs of 7, 9 and 10 scale number contribute less to fault classification. Meanwhile, to avoid excess information loss only the first three PE features with small importance value were removed from the original MPE features. As the estimation results presented in Figure 9b, the PEs of 5, 7 and 8 scale number are eliminated in Case 2.

In order to verify the effectiveness of OOB estimation in features evaluation, the refined VMD-PE features, as shown in Figure 9, were employed to train the 2nd-RF based diagnosis model and the diagnosed results are displayed in Table 3. As can be seen from this table, the average diagnosis accuracy of the method with OOB estimation in the fault identification stage is 98.48% (100%−1.52%) and 99.97% (100%−0.3%), respectively, and the result of the method with the original VMD-PE features is 98.44% and 99.09%. Obviously, only a slight improvement in diagnosis accuracy can be obtained by using OOB estimation, but the refined features are more simple and sensitive and result in high diagnosis efficiency. With a slight improvement of accuracy in the second step, a certain enhancement of the final diagnosis accuracy can also be achieved, as shown in the 4th column and the 7th column of Table 3. Meanwhile, the proposed method was compared with the one-step method. By using the one-step model, the whole VMD-PE features dataset was divided into two groups: 25% and 75%. The 25% of samples are training set and the rest of the samples are testing samples. The diagnosis results obtained by the one-step method are listed in the fourth row of Table 3. As for the average diagnosis accuracy, the proposed method also outperforms the one-step method. These comprehensive results prove that the proposed method could classify the test samples efficiently and ultimately complete the fault diagnosis of rolling element bearings.

## 5. Conclusions

In this study, a two-step fault diagnosis scheme based on permutation entropy with different scales and random forests is proposed for rolling element bearings. In the fault detection step, a preliminary judgment about the health condition of bearings can be easily achieved with 100% accuracy, as shown in Figure 5 and Figure 6. By using this statistical classification model based on Chebyshev’s inequality and PE, the subsequent workload in the next step can be reduced substantially and it can help workers to realize the real time monitoring of equipment. In order to further confirm the fault information of bearings, a RF-based diagnosis model based on VMD-PE features and OOB estimation is established. The results of Figure 8 and Table 2 show that VMD-PE features can effectively reveal the dynamic characteristics of vibration signals with different fault conditions and RF is more applicable to the intelligent fault diagnosis of bearings. Furthermore, this is the first time that the OOB estimation is used to evaluate the features importance and the results of Table 3 demonstrate its availability and practicality. Finally, the proposed method is also contrasted with the single step diagnosis model. The comparison results have verified the advantages of the proposed method in accuracy and the proposed diagnosis strategy is more in line with fault evolvement rule and human perception.

However, the applications of RF in fault diagnosis are still at an early stage, or else its diagnosis performance may be further improved. For example, its parameters often are determined by experience values, and in some real applications, the experience values are not suitable. Hence, the parameters optimization of RF is our next research point. In addition, the PE value of the signals with early weak or serious defect is probably closer to the value of normal signals, resulting in misdiagnosing these defects as normal. Therefore, how to establish a sounder diagnosis model with multiple features fusion is also our next study focus.

## Figures and Tables

**Table 1:** Twelve fault conditions of bearings under loads of 0 hp (Case 1) and 2 hp (Case 2).

	Type	Inner Race Fault	Outer Race Fault	Ball Fault	Normal
Size (cm)					
**0.0178**		√	√	√	√
**0.0356**		√	√	√	
**0.0533**		√	√	√	
**0.0711**		√	--	√	
“√” indicates the working condition is under consideration.					

**Table 2:** Comparison of diagnosis results obtained by different classifiers and VMD-PE features.

	Case 1		Case 2	
	Accuracy (%)	Cost Time (s)	Accuracy (%)	Cost Time (s)
MPE-ELM ^1^	94.11 ± 1.11	0.006	96.15 ± 1.45	0.007
MPE-SVM	96.76 ± 0.86	5.452	97.83 ± 1.01	5.029
MPE-RF	**98.44 ± 0.67**	**0.075**	**99.09 ± 0.67**	**0.074**

^1^ MPE, multiscale permutation entropy; ELM, extreme learning machine; SVM, support vector machine; RF, random forests.

**Table 3:** Diagnosis results obtained by the proposed method, the two-step method with no features refinement and the traditional one-step method. OOB, out-of-bag.

	Case 1			Case 2		
	*E*_1_ (*H* = 350)	*E*_2_ (*h* = 330)	*η*	*E*_1_ (*H* = 350)	*E*_2_ (*h* = 330)	*η*
Two-step+ OOB	0%	1.52%	**98.57%**	0%	0.30%	**99.997%**
Two-step	0%	1.56%	**98.57%**	0%	0.91%	**99.14%**
One-step	-	-	**97.50%**	-	-	**98.89%**
